# Antioxidant Activity and Antibacterial Effects on Clinical Isolated *Streptococcus suis* and *Staphylococcus intermedius* of Extracts from Several Parts of *Cladogynos orientalis* and Their Phytochemical Screenings

**DOI:** 10.1155/2015/908242

**Published:** 2015-08-11

**Authors:** Pongtip Sithisarn, Piyanuch Rojsanga, Patchima Sithisarn, Sumet Kongkiatpaiboon

**Affiliations:** ^1^Department of Pharmacognosy, Faculty of Pharmacy, Mahidol University, Bangkok 10400, Thailand; ^2^Department of Pharmaceutical Chemistry, Faculty of Pharmacy, Mahidol University, Bangkok 10400, Thailand; ^3^Department of Veterinary Public Health, Faculty of Veterinary Medicine, Kasetsart University, Nakhon Pathom 73140, Thailand; ^4^Drug Discovery and Development Center, Thammasat University, Rangsit Campus, Pathum Thani 12121, Thailand

## Abstract

The *in vitro* antioxidant and antibacterial assays against clinically isolated *Streptococcus suis* and *Staphylococcus intermedius* of the extracts prepared by decoction and ethanolic reflux of different parts of Chettaphangki (*Cladogynos orientalis* Zipp. ex Span), including the leaves, roots, and stems, using 1,1-diphenyl-2-picrylhydrazyl (DPPH) scavenging assay and disc diffusion method were conducted. Quantitative analysis of total phenolic and total flavonoid contents in the extracts using spectrophotometric methods was also performed. Finally, phytochemical screening by thin layer chromatography (TLC) and high performance liquid chromatography (HPLC) was conducted. Leaf ethanolic reflux extract (100 g) contained the highest total phenolic and total flavonoid contents of 7.21 ± 0.28 *μ*g gallic acid equivalent (GAE) and 11.51 ± 2.02 *μ*g rutin equivalent (RE), respectively. Chettaphangki extracts promoted low antioxidant activity with EC_50_ values in the range of 0.27–0.48 mg/mL. Extracts and fractions from the roots and stems of this plant promoted low to intermediate antibacterial activity against *S. intermedius* with the inhibition zones between 7 and 14 mm. The chromatographic data suggested that the leaf extracts of *C. orientalis* contained rutin while the root and stem extracts contained scopoletin and chettaphanin I. Rutin promoted strong antioxidant activity while chettaphanin I showed low antibacterial activity against *Staphylococcus intermedius*.

## 1. Introduction

*Cladogynos orientalis*, colloquially known in Thai as Chettaphangki, is a plant in Euphorbiaceae family which has been used in traditional medicine as antiflatulent, antistomachache, and tonic agent [1]. The roots of this plant are assigned in National List of Essential Medicines 2013, as the component in the formulation to treat flatulence and colic [1]. Previous report suggested that the extract from the whole plant of* C. orientalis* promoted antidengue virus effect using MTT assay [2]. The leaf extract also promoted effective inhibition of human hepatocarcinoma (HepG2) [3]. It was previously reported that terpenoid compounds including ent-halimane diterpenes; chettaphanin I and chettaphanin II, sesquiterpenes; 8-hydroxy-alpha-guaiene, spathulenol, cyperenoic acid, and triterpenes; and taraxerol and acetoxyaleuritolate were the major components in* C. orientalis* [4–6]. Some unusual aromatic diglycosides including 4′′-O-galloyl-violutoside and 4′′-O-galloyl-benzyl-O-*α*-L-rhamnopyranosyl-(1→6)-*β*-D-glucopyranoside and some flavonoids such as isovitexin and apigenin glycosides were found in the aerial part of this plant [7]. Moreover, the leaves were also reported to contain some phenolics and flavonoids including chlorogenic acid, epicatechin, and quercetin [3]. Despite reports suggesting some biological activities of* C. orientalis* extract, the ethnomedical use of this plant as tonic and agent to treat flatulence and stomachache is yet to be investigated. Therefore, this experiment was set up in order to screen for antioxidant and antibacterial activities against* Streptococcus suis* and* Staphylococcus intermedius* of extracts from various parts of* C. orientalis* prepared by decoction and reflux with ethanol. After that, quantitative analysis of total phenolic and total flavonoid contents and phytochemical study of the extracts using spectrophotometric and chromatographic techniques were also conducted.

## 2. Materials and Methods

### 2.1. Plant Extracts Preparation

Several parts of* Cladogynos orientalis* including the leaves, stems, and roots were purchased from Muang District, Nakhon Phanom Province, Thailand, in October 2013. Plant samples were identified by Professor Dr. Wongsatit Chuakul, Department of Pharmaceutical Botany, Faculty of Pharmacy, Mahidol University, Bangkok, Thailand. All samples were cleaned and dried in hot air oven (50°C) for 6 h and powdered by electronic mill (20-mesh sieve). Each sample was extracted using procedures below.

*Decoction.* Dried powder of each sample was separately boiled (80°C) with distilled water (plant/water ratio 1 : 10 w/v) for 3 h and then filtered. The filtrate was dried using water bath to obtain dried plant decoction extracts, namely, leaf decoction extract (COLD), root decoction extract (CORD), and stem decoction extract (COSD).

*Reflux.* Dried powder of each sample was separately refluxed with 75% ethanol (plant/water ratio 1 : 10 w/v) for 3 h and then filtered. The filtrate was dried using water bath to obtain dried plant ethanol extracts which were leaf reflux extract (COLE), root reflux extract (CORE), and stem reflux extract (COSE).

The roots of* C. orientalis* promoted the extracts with antioxidant activity and contained some phenolics and flavonoids. They also promoted specific chromatographic fingerprints with the presence of some interesting phytochemicals. Therefore the roots of* C. orientalis* were selected for further extraction by various methods including decoction with freeze dry and Soxhlet extraction with 95% ethanol. Moreover, solvent-solvent extraction of root decoction with freeze dried extract was performed using distilled water and dichloromethane.

*Decoction with Freeze Drying.* Dried root powder of* C. orientalis* was boiled (80°C) with distilled water (plant/water ratio 1 : 10 w/v) for 3 h and then filtered. The extraction process was repeated twice. The filtrates were combined and dried using freeze dry machine (SciQuip Ltd., UK) to obtain dried root decoction with freeze dried extract (CORDF).

*Soxhlet Extraction with 95% Ethanol.* Dried root powders of* C. orientalis* were extracted with 95% ethanol (plant/water ratio 1 : 10 w/v) using Soxhlet apparatus at 70°C until being exhausted (28 h). The extract solution was dried on a water bath to obtain dried root Soxhlet extraction extract (COREX).

*Solvent-Solvent Extraction of CORDF.* Ten grams of dried root decoction with freeze dried extract (CORDF) was fractionated using solvent-solvent extraction technique with distilled water and dichloromethane (extract/each solvent ratio 1 : 10 w/v) for 30 min. The fractionation process was repeated twice. The aqueous and dichloromethane fractions were separately combined. Each fraction was dried on a water bath to yield dried aqueous fraction from root decoction with freeze drying extract (CORDF (AQ)) and dichloromethane fraction from root decoction with freeze drying extract (CORDF (DCM)).

### 2.2. Determination of Total Phenolic Content Using Folin-Ciocalteu Method [8]

Plant extract solutions (25 *μ*L) were oxidized with Folin-Ciocalteu reagent (25 *μ*L) in 96-well plate; then 75 *μ*L of distilled water and 100 *μ*L of 20% sodium carbonate solution were added. The absorbance of the resulting blue colored solution was measured at 765 nm after 60 min using Microplate Reader (Tecan, USA). Each sample was done in triplicate. Total phenolic content was calculated from standard curve of gallic acid and was expressed as mg gallic acid equivalent in 100 g extract (mg% GAE).

### 2.3. Determination of Total Flavonoid Content [9]

Plant sample solutions (100 *μ*L) were separately reacted with 2% aluminium chloride solution in the same volume. The absorbance was read at 415 nm after 10 min using Microplate Reader (Tecan, USA). Flavonoid content was calculated from standard curve of rutin and was expressed as milligram rutin equivalent in 100 mg of plant extracts (mg% RE).

### 2.4. Determination of Antioxidant Activity by DPPH Scavenging Assay [10]

The free radical scavenging activities of plant extracts and of standard solution (ascorbic acid) were investigated using 1,1-diphenyl-2-picrylhydrazyl (DPPH) radical scavenging method. A total of 100 *μ*L of the extract or standard was added to 100 *μ*L of DPPH in methanol solution (152 *μ*M). After staying at room temperature for 15 min, the absorbance of each solution was determined at 517 nm using Microplate Reader (Tecan, USA). The percentage of inhibition was calculated; then the EC_50_ value, the concentration of sample required for 50% scavenging of the DPPH free radical, was determined. Each determination was done in triplicate, and the average EC_50_ value was calculated.

### 2.5. Antibacterial Activity Determination

#### 2.5.1. Bacteria and Reagents

Clinically isolates of* Streptococcus suis* (3 isolates) and* Staphylococcus intermedius* (3 isolates) were obtained from Microbiological Laboratory, Veterinary Diagnostic Center, Faculty of Veterinary Medicine, Kasetsart University, Nakhon Pathom, Thailand. The bacteria strains were isolated and characterized by the differential bacterial culture and biochemical assays for clinical samples according to the standard method of Baron et al. (1994) [11].* S. suis* were maintained in Microbank Cryovials and kept at −80°C.* Staphylococcus intermedius* were maintained in skimmed milk at −40°C until being used. Blood agar was obtained for Bacteriology Unit. Antibiotic discs, Amoxicillin/clavulanic acid 30 *μ*g, Doxycycline 30 *μ*g, and Sulfa-trimethoprim 25 *μ*g were purchased from Oxoid, UK. Fosfomycin 50 *μ*g was purchased from Bedson Co., Argentina. Chettaphanin I 10 mg/mL was used as control standard.

#### 2.5.2. Bacteria Culture

Prior to sensitivity testing, each bacterial strain was cultured on blood agar plate and incubated for 18–24 h at 37°C. A single colony was then cultured in 5 mL Mueller-Hinton (MH) Broth for 4 hours at 37°C. The density of bacteria culture required for the test was adjusted to 0.5 McFarland standard, (1.0 × 10^8^ CFU/mL) using the Turbidimeter (BioSan, Latvia).

#### 2.5.3. Plant Extracts Dilution and Preparation of Impregnated Disc

Ten dried extract candidates from several parts of* Cladogynos orientalis* prepared by different methods of extraction with satisfying antioxidant activities were submitted for the antibacterial property. All extracts were prepared for antibacterial screening assays at the concentration of 10 mg/mL. The extracts were dissolved in their solvents of origin (70% ethanol or distilled water) and stored at 4°C for further use.

#### 2.5.4. Disc Diffusion Method

Disc diffusion method for antimicrobial susceptibility testing was carried out according to the standard method by Bauer et al. (1966) [12] to assess the presence of antibacterial activities of the plant extracts. A bacteria culture was inoculated to the entire surface of Mueller-Hinton agar plate using sterile swab. The plates were dried for 15 minutes and used for the sensitivity assay. The discs were infused with 10 *μ*L (100 *μ*g) plant extract/disc and placed on the Mueller-Hinton agar surface. Each test set comprises ten implanted extract discs, antibiotic controls, chettaphanin I, and negative controls. The standard antibiotic discs were Amoxicillin/clavulanic acid 30 *μ*g, Doxycycline 30 *μ*g, and Sulfa-trimethoprim 25 *μ*g for* S. intermedius.* Amoxicillin/clavulanic acid 30 *μ*g, Doxycycline 30 *μ*g, and Fosfomycin 50 *μ*g discs were for* S. suis*. The negative controls, ethanol and water, were saturated onto the blank disc (Oxoid, UK) and applied along the test. Each test plate had four treated discs placed in equidistance to each other. The plates were incubated at 37°C for 24 hours and examined for zone of inhibition (ZOI). Three replicates were carried out for each bacteria isolate. Data were expressed as mean ± standard deviation from three isolates of* S. suis* and* S. intermedius*.

### 2.6. Phytochemical Screenings

#### 2.6.1. Thin Layer Chromatographic (TLC) Fingerprints

Thin layer chromatography of all extracts was performed on TLC precoated silica gel 60 GF254 plate using hexane-ethyl acetate-formic acid (7 : 3 : 0.1) as solvent system. TLC plates were detected under UV 254 and 366 nm and NP/PEG under UV 366 nm and anisaldehyde sulfuric acid spraying reagents.

#### 2.6.2. High Performance Liquid Chromatographic Fingerprint

Analysis of phytochemicals in extracts from various parts of* C. orientalis* was performed with Agilent 1260 series equipped with UV diode array detector. A Hypersil BDS-C_18_ column (4.6 mm i.d. × 15 cm, 3.5 *μ*m) was used for quantitative analysis. Gradient elution was performed with 0.5% acetic acid in water (solvent A) and methanol (solvent B) at constant flow rate of 1 mL/min. The gradient program was adjusted from 0% to 100% B in 40 min and stayed at 100% B for 10 min. The column was equilibrated with 100% A for 10 min prior injection. Column temperature was 25°C with an injection volume of 10 *μ*L. UV detection was performed at 254 and 310 nm.

### 2.7. Statistical Analysis

All data are reported as means ± standard deviation of triplicates analysis. Least significant difference was used to compare means (*P* < 0.05). All analyses were performed using SPSS for Windows, version 16.0 (SPSS Inc., USA).

## 3. Results and Discussion

### 3.1. Determination of Total Phenolic and Total Flavonoid Contents

As shown in Table 1,* C. orientalis* leaf ethanol reflux extract (COLE) significantly exhibited the highest total phenolic and total flavonoid contents of 7.21 *μ*g GAE and 11.51 *μ*g RE in 100 g extract, respectively. Even though there was no report concerning phenolic and flavonoid contents in this plant before, the amounts of phenolic and flavonoid contents in all* C. orientalis* extracts found in this experiment were quite low compared to the report of total phenolic and total flavonoid contents in some Thai indigenous plants [13].

### 3.2. Determination of Antioxidant Activity

All* C. orientalis* extracts exhibited low free radical scavenging effects on DPPH radicals as shown in Table 1. The EC_50_ values of all extracts ranged from 0.27 to 0.48 mg/mL where the stem refluxing extract (COSE) promoted the strongest antioxidant activity among the tested samples. The results supported the previous report of the extract from* C. orientalis* promoting low* in vitro* antioxidant effects determined by Folin and TEAC assay [14]. Moreover, there was a study that suggested that the EC_50_ values of DPPH scavenging test for antioxidant activities of some Thai medicinal plants ranged from 0.06 to 15.20 mg/mL [15]. From the results, there was no correlation between the amounts of total phenolic and total flavonoid contents and DPPH scavenging activity of the extracts from* C. orientalis*.

### 3.3. Antibacterial Activity Determination

The antibacterial activities of extracts against six clinical isolates of* S. suis* and* S. intermedius* were determined by the diameter of inhibition zones as shown in Table 2. Six of the ten extracts mostly from the roots and stems of this plant, root reflux extract (CORE), stem reflux extract COSE, root Soxhlet extraction extract COREX, root decoction with freeze dried extract (CORDF) and aqueous fraction from root decoction with freeze dried extract (CORDF (AQ)), and dichloromethane fraction from root decoction with freeze dried extract (CORDF (DCM)), promoted low to intermediate antibacterial activity against* S. intermedius* with the inhibition zones between 7 and 14 mm. Nonetheless, no antibacterial activity was established from any parts of* C. orientalis* against* S. suis*. Broad but low antibacterial activity of the root ethanol Soxhlet extract (COREX) was observed as three isolates of* S. intermedius* showed sensitivities. Intermediate activity of CORDF (DCM) was shown in one strain of* S. intermedius*. Regarding the antioxidant activity, there is no correlation between the amounts of total phenolic and total flavonoid and antibacterial activity of the extracts from* C. orientalis*. However, stem reflux extract (COSE) which showed the highest antioxidant activity also promoted inhibitory effect on* S. intermedius*. It has been revealed that antioxidants interfered oxidative status and mediated reactive oxygen species (ROS) in bacterial infections [16]. In* S. suis*, superoxide dismutase (SOD) is suggested to be one of the virulent factors possibly by increasing resistance to oxidative stress [17]. Though the role of SOD for* S. suis* pathogenesis is still unclear, recent report from Fang et al., 2015 [18], showed that* S. suis* required SOD to scavenge ROS for survival in infected macrophages. Therefore, it is suggested that COSE and also other* C. orientalis* extracts affected bacterial oxidative status and/or metabolism of reactive oxygen species which may involve in SOD functions.

Another possibility is the interference of bacterial biofilm formation probably in one or more of the developmental steps of cellular adhesion, maturation, and signaling. Many flavonoids have been reported to possess antibacterial activity by disturbing bacterial adhesion, microcolony formation, and bacterial quorum sensing [19, 20]. Complex molecular signaling proteins and cellular receptors are reported to be involved [21, 22]. However, the mechanisms of the substances on bacterial biofilm formation and quorum sensing are not yet well explained. The studies on the extracts and phytochemicals of* C. orientalis* against* S. suis* and* S. intermedius* may provide valuable information on the mechanism of flavonoids' antibacterial activity.

*S. suis* and* S. intermedius* are important pathological bacteria in animal species that can cause human encephalitis. It would be interesting to test the six potent extracts in a higher concentration than 100 *μ*g against these bacteria. In addition, due to the traditional uses of* C. orientalis* for treatment of flatulence and stomachache, other pathological bacteria, especially the ones that cause respiratory and gastrointestinal diseases such as* Escherichia coli*,* Pseudomonas aeruginosa*, and* Salmonella*, should be subjected to this determination.

### 3.4. Phytochemical Screenings

#### 3.4.1. TLC Fingerprints

**Figure 1:**
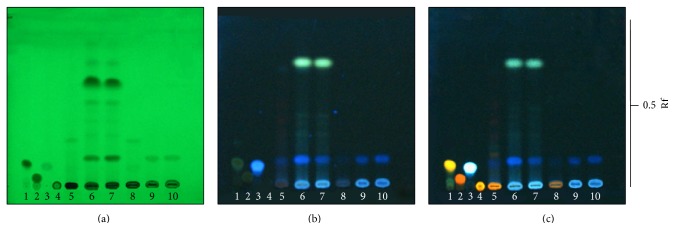
TLC chromatogram of* C. orientalis* extracts; 1 = quercetin, 2 = myricetin, 3 = caffeic acid, 4 = rutin, 5 = COLE, 6 = COSE, 7 = CORE, 8 = COLD, 9 = COSD, and 10 = CORD. Adsorbent: silica gel GF254. Solvent system: hexane-ethyl acetate-formic acid (7 : 3 : 0.1). Detection: (a) UV 254 nm, (b) UV 366 nm, and (c) NP/PEG under UV 366 nm. Band identification: rutin (Rf = 0.01).

**Figure 2:**
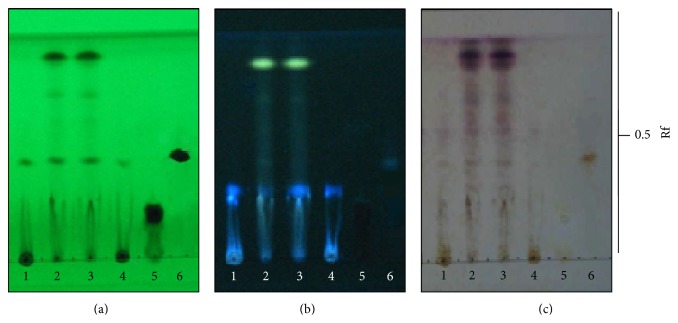
TLC chromatogram of* C. orientalis* extracts; 1 = CORD, 2 = CORE, 3 = COSE, 4 = COSD, 5 = rutin, and 6 = chettaphanin I. Adsorbent: silica gel GF254. Solvent system: hexane-ethyl acetate-formic acid (7 : 3 : 1). Detection: (a) UV 254 nm, (b) UV 366 nm, and (c) anisaldehyde sulfuric acid. Band identification: chettaphanin I (Rf = 0.45).

All extracts exhibited chromatographic TLC fingerprints as shown in Figure 1. The leaf decoction and ethanol reflux extracts produced chromatographic bands that corresponded to some phenolics and flavonoids. The chromatographic band analyzed by TLC at the Rf values of 0.01 was identified as rutin. Moreover, it was found that the stem and root extracts from both decoction and ethanol reflux methods contained the chromatographic bands that corresponded to chettaphanin I (Rf = 0.45) as shown in Figure 2.

#### 3.4.2. HPLC Fingerprints

**Figure 3:**
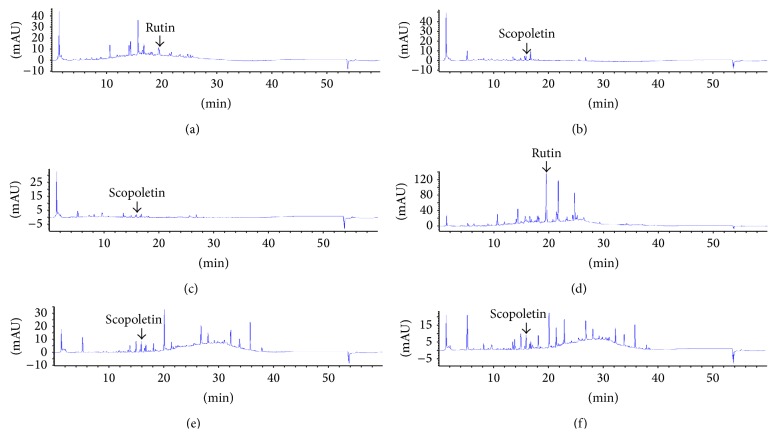
HPLC chromatographic fingerprints from the leaves, roots, and stems of* Cladogynos orientalis* prepared by two different methods including decoction and refluxing with 75% ethanol. (a) COLD, (b) CORD, (c) COSD, (d) COLE, (e) CORE, and (f) COSE. Column: Hypersil BDS C18. Mobile phase: water/0.5% acetic acid (solvent A) and methanol (solvent B); gradient. Detector: photodiode array detector at 310 nm. Peak identification: scopoletin (Rt = 15.95 min) and rutin (Rt = 19.57 min).

**Figure 4:**
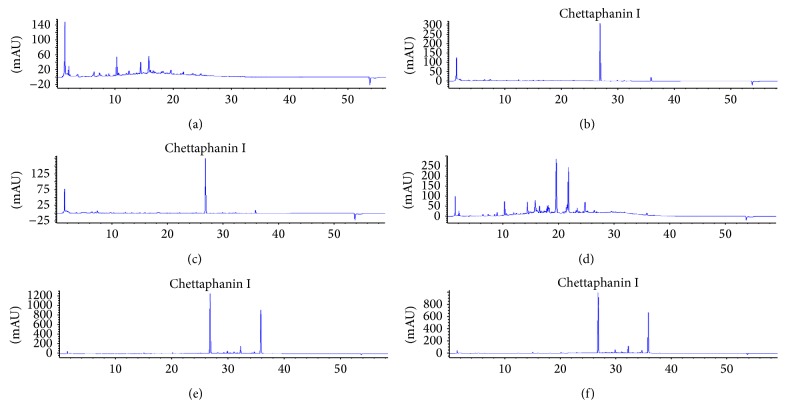
HPLC chromatographic fingerprints from the leaves, roots, and stems of* Cladogynos orientalis* prepared by two different methods including decoction and refluxing with 75% ethanol. (a) COLD, (b) CORD, (c) COSD, (d) COLE, (e) CORE, and (f) COSE. Column: Hypersil BDS C18. Mobile phase: water/0.5% acetic acid (solvent A) and methanol (solvent B); gradient. Detector: photodiode array detector at 254 nm. Peak identification: chettaphanin I (Rt = 26.82 min).

HPLC analyses of the crude extract from the leaves, roots, and stem of* C. orientalis* were carried out with photodiode array detector. The results revealed that all extracts promoted specific chromatographic fingerprints. Identification of peaks was done by comparing with standards rutin (Sigma-Aldrich, USA), scopoletin (Tokyo Chemical Industry, Japan), and chettaphanin I (authentic standard, separated and identified by Assistant Professor Dr. Pongtip Sithisarn and Dr. Sumet Kongkiatpaiboon). In comparison of the retention times and their UV absorption spectra with those of standards, the leaf extracts from* C. orientalis* contained rutin (Rt = 19.57) as shown in Figure 3, while the root and stem extracts contained scopoletin (Rt = 15.95) and chettaphanin I (Rt = 26.82) as shown in Figure 4. From our experiment using DPPH scavenging assay, it was found that rutin which is the flavonoid presented in the leaf extracts promoted strong antioxidant activity while scopoletin and chettaphanin I promoted low antioxidant activity (Table 1).

From phytochemical analysis, it was found that the roots and stems of* C. orientalis* contained chettaphanin I which supported the previous studies [4]. This compound was found to promote mild inhibitory effect on* Mycobacterium tuberculosis* [23]. However, this is the first report of the* in vitro* antioxidant and antibacterial activities of this compound. Moreover, this is also the first report on the presence of scopoletin in the roots and stems of* C. orientalis* and rutin in the leaves of this plant. Since* C. orientalis* is officially listed in the National List of Essential Medicines 2013 of Thailand, the developed analytical methods by TLC and HPLC from this experiment could be applied for quality control of raw material and extracts from several parts of this plant. The identified compounds which are rutin, scopoletin, and chettaphanin I could be used as markers for standardization and quality control process in the future.

## 4. Conclusion

*C. orientalis* extracts promoted low* in vitro* antioxidant activity as determined by the DPPH scavenging assay. Root reflux, stem reflux, root Soxhlet extraction, root decoction with freeze dried extracts, and aqueous and dichloromethane fractions from root decoction with freeze dried extract showed low to intermediate antibacterial activity against* Staphylococcus intermedius.* None of the extracts showed activity against* Streptococcus suis*. All extracts contained low amounts of total phenolic and total flavonoid. The leaf extracts of* C. orientalis* contained rutin while the root and stem extracts contained scopoletin and chettaphanin I. Rutin promoted strong antioxidant activity while chettaphanin I showed low antibacterial activity against* Staphylococcus intermedius*.

## Figures and Tables

**Table 1:** Total phenolic and total flavonoid contents and *in vitro* antioxidant activity of extracts from various parts of *C. orientalis*.

Sample	Yield (%w/w)	Total phenolic content (*μ*g GAE/100 g extract)	Total flavonoid content (*μ*g RE/100 g extract)	Antioxidant activity (EC_50_, mg/mL)
COLD	9.22	5.86 ± 0.38^a^	5.20 ± 0.85^a^	0.35 ± 0.00^a^
CORD	5.52	6.05 ± 0.53^a^	4.82 ± 0.47^a^	0.34 ± 0.01^a^
COSD	2.26	3.60 ± 0.14^b^	3.15 ± 0.60^a^	0.43 ± 0.01^b^
COLE	3.10	7.21 ± 0.28^c^	11.51 ± 2.02^b^	0.48 ± 0.01^c^
CORE	4.50	3.30 ± 0.25^b^	2.84 ± 0.88^a^	0.31 ± 0.01^d^
COSE	4.42	3.57 ± 0.09^b^	3.78 ± 1.52^a^	0.27 ± 0.01^e^
Ascorbic acid		—	—	2.53 ± 0.42 × 10^−3^
Rutin		—	—	6.48 ± 0.18 × 10^−3^
Scopoletin		—	—	0.19 ± 0.01
Chettaphanin I		—	—	2.40 ± 0.19

^*∗*^Different letters in the same column are significantly different (*P* < 0.05).

**Table 2:** Antibacterial activities of *C. orientalis* extracts to *Streptococcuss suis* and *Staphylococcus intermedius *by disc diffusion assays.

Sample	Zone of inhibition (mean ± SD mm)					
	*S. suis *isolates			*S. intermedius *isolates		
	1	2	3	1	2	3
COLD	0	0	0	0	0	0
CORD	0	0	0	0	0	0
COSD	0	0	0	0	0	0
COLE	0	0	0	0	0	0
CORE	0	0	0	8.33 ± 1.15	0	11.00 ± 0.00
COSE	0	0	0	8.00 ± 1.00	0	11.33 ± 1.15
COREX	0	0	0	9.00 ± 1.00	7.67 ± 0.58	11.33 ± 0.58
CORDF	0	0	0	8.00 ± 1.73	0	9.67 ± 1.15
CORDF (AQ)	0	0	0	8.67 ± 0.57	0	10.67 ± 0.58
CORDF (DCM)	0	0	0	8.00 ± 1.00	0	14.33 ± 0.58
Chettaphanin I	0	0	0	9.67 ± 0.57	0	7.00 ± 0.00
DO30	15.33 ± 0.58	18.00 ± 0.00	21.67 ± 1.53	18.33 ± 1.15	13.67 ± 0.58	17.33 ± 0.58
AMC30	13.33 ± 1.15	28.00 ± 0.00	38.00 ± 2.00	30.67 ± 1.15	13.67 ± 0.58	26.67 ± 1.15
SXT25	NA	NA	NA	10.00 ± 1.00	8.67 ± 0.58	11.00 ± 1.00
FOSBAC	23.67 ± 1.15	30.00 ± 0.00	20.00 ± 0.00	NA	NA	NA

AMC30 = Amoxicillin/clavulanic acid 30 *μ*g, DO30 = Doxycycline 30 *μ*g, SXT25 Sulfa-trimethoprim 25 *μ*g, FOSBAC = fosfomycin 50 *μ*g. NA = not applicable, selection due to the sensitivity of microbes to reference antibiotics.
